# LiNbO_**3**_ Coating on Concrete Surface: A New and Environmentally Friendly Route for Artificial Photosynthesis

**DOI:** 10.1155/2013/686497

**Published:** 2013-11-25

**Authors:** Ranjit K. Nath, M. F. M. Zain, Abdul Amir H. Kadhum

**Affiliations:** ^1^Sustainable Construction Materials and Building Systems (SUCOMBS) Research Group, Faculty of Engineering and Built Environment, Universiti Kebangsaan Malaysia, 43600 Bangi, Malaysia; ^2^Department of Chemical & Process Engineering, Faculty of Engineering and Built Environment, Universiti Kebangsaan Malaysia, 43600 Bangi, Malaysia

## Abstract

The addition of a photocatalyst to ordinary building materials such as concrete creates environmentally friendly materials by which air pollution or pollution of the surface can be diminished. The use of LiNbO_3_ photocatalyst in concrete material would be more beneficial since it can produce artificial photosynthesis in concrete. In these research photoassisted solid-gas phases reduction of carbon dioxide (artificial photosynthesis) was performed using a photocatalyst, LiNbO_3_, coated on concrete surface under illumination of UV-visible or sunlight and showed that LiNbO_3_ achieved high conversion of CO_2_ into products despite the low levels of band-gap light available. The high reaction efficiency of LiNbO_3_ is explained by its strong remnant polarization (70 **µ**C/cm^2^), allowing a longer lifetime of photoinduced carriers as well as an alternative reaction pathway. Due to the ease of usage and good photocatalytic efficiency, the research work done showed its potential application in pollution prevention.

## 1. Introduction

In the last two centuries fossil fuels and the cheap energy have been provided for energy in the industrial development [1]. The excessive use of fossil fuels has produced CO_2_ in the earth's atmosphere which is one of the responsible for greenhouse effect [2]. The effect of increasing atmospheric carbon dioxide is directly linked to global warming [3]. The reduction of CO_2_ by the process of artificial photosynthesis is believed to be one of the alternatives that emits oxygen using atmospheric CO_2_ and water in presence of photocatalyst [2]. 

Artificial photosynthesis was investigated by using a variety of approaches [4] like replication of the chemical reactions that take place in plants [5] and concerted efforts using ruthenium complexes and structures as an organic dye [6] to sensitize wide band-gap semiconductors in presence of visible light [7]. In this process a semiconductor is used as a photocatalyst that can absorb light and using this energy, it can chemically convert CO_2_ and water into formic acid and oxygen [8, 9]. In this work, we describe artificial photosynthesis taking place on photocatalyst LiNbO_3_ coated on concrete surface by producing formic acid and oxygen.

## 2. Approach to Artificial Photosynthesis

**Figure 1:**
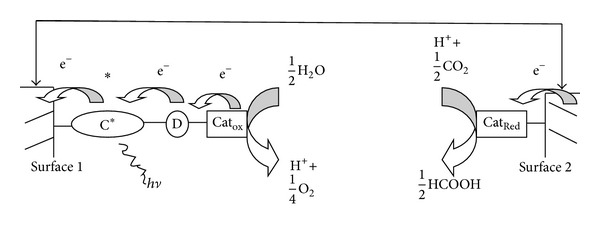
Schematic diagram illustrating the photoelectrochemical reaction between CO_2_ and H_2_O to give HCOOH and O_2_ initiated by excitation and photoinjection in presence of photocatalyst.

The goal of artificial photosynthesis is to mimic the green plants and other photosynthetic organisms that use sunlight to make valuable substances [10]. In natural photosynthesis, the first step is water splitting in which proton is generated and O_2_ is released using solar energy [11]. The second step is the Calvin cycle in which CO_2_ is reduced to hydrocarbons [4]. Schematic diagram is shown in Figure 1. This is a challenging goal because success requires integration of multiple chemical functions in stable chemical architecture. 

### 2.1. Catalyst for CO_2_ Reduction

To perform artificial photosynthesis, the photocatalyst must be able to drive the reduction of CO_2_ and, ideally, the oxidation of water [12]. To achieve this, the reaction potentials must be inside the band edges of the photocatalyst [13]. Besides many tested semiconductors (ZnO, Fe_2_O_3_, CdS, ZnS, and TiO_2_), LiNbO_3_ can quickly reduce CO_2_ more than others [14–16].

**Figure 2:**
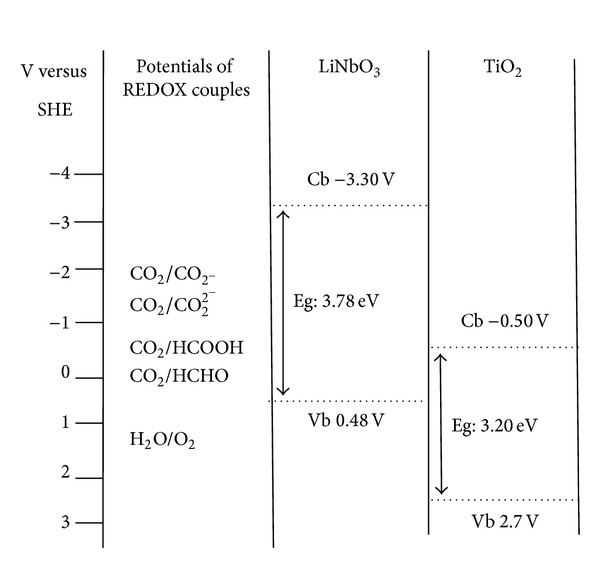
Band positions of LiNbO_3_ and TiO_2_ versus SHE in relation to the redox reactions of artificial photosynthesis [7].

Figure 2 compares the potential of the valence and conduction bands of LiNbO_3_ and mostly used photocatalyst TiO_2_ in relation to the cell potential of the conversion of CO_2_ [17]. This figure shows that in TiO_2_ valence band holes can oxidize water and conduction band electrons cannot reduce CO_2_ to CO_2_^−^ due to low energy than required Table 1. LiNbO_3_ drives the reaction of CO_2_ and water to formic acid, but the valence band holes do not oxidize water directly [8]. This is due to its valence band and conduction band energy level. The valence and conduction band energy of LiNbO_3_ are 0.48 v and −3.30 v. Conduction band electrons of LiNbO_3_ can reduce CO_2_ to CO_2_^−^ due to high energy and valence band holes oxidize water by physisorbing due to its strong remnant polarization [8]. The reaction step of LiNbO_3_ allows a different reaction pathway because of its highly reducing conduction band electrons [7]. 

### 2.2. Water Oxidation Catalyst for Artificial Photosynthesis

Water oxidation is a more complex chemical reaction than proton reduction. In natural photosynthesis, the oxygen-evolving complex performs this reaction by accumulating reducing equivalents (electrons) in a manganese-calcium cluster within photosystem II (PS II) and then delivers them to water molecules [18], with the resulting production of molecular oxygen and protons:

(1) $$\begin{array}{c} 2{\text{H}}_{2}\text{O}\to {\text{O}}_{2}+4{\text{H}}^{+}+4{\text{e}}^{-} \end{array}$$

Without a catalyst (natural or artificial), this reaction is very endothermic and it requires high temperatures (at least 2500 K) [19]. Many metal oxides have been found to have water oxidation catalytic activity [20, 21], especially those from relatively abundant transitional metals, but these metal oxides suffer from low turnover frequency and slow electron transfer properties, and their mechanism of action is hard to decipher and adjust [8].

Remnant polarization of LiNbO_3_ allows this process at different reaction pathway that leads to a charge occurring on the interface [22]. This charge interacts with species in contact with the surface, producing a tightly bound layer [23–25] that changes the nature of bonding in physisorbed materials. In the case of water, LiNbO_3_ will physisorb a tight layer of molecules [26]. The valence band location of LiNbO_3_ (0.48 V versus SHE) is unable to oxidize water directly; the physisorbed water appears to prevent any further reactions on the surface of the catalyst [27]. In the gas-solid reaction, there is more CO_2_ available and bound on the surface of the LiNbO_3_ [8]. This bound CO_2_ reacts with photoexcited holes and electrons in the LiNbO_3_ and produces reactive species that react with H_2_O to form end products [10]. One such species is CO_2_^−^ that reacts with water to produce formic acid and oxygen:

(2) $$\begin{array}{c} {\text{H}}_{2}\text{O}+{{\text{CO}}_{2}}^{-}\to \text{HCOOH}+\frac{1}{2}{\text{O}}_{2}+{\text{e}}^{-} \end{array}$$

The physisorption of molecules on ferroelectric surfaces [27] affects the localization of electrons in the molecule and the bond angles. This condition allows for easier injection or removal of electrons than that in less chemically strained systems. 

## 3. Materials and Methods

### 3.1. Materials

Ordinary portland cement, sand, and stone aggregate are used to prepare the concrete sample commercially available in Malaysia. In this research locally available coarse river sand and stone aggregate of 10 mm size were used. The specific gravity of sand and aggregate used in this research is 2.63 and 2.65, respectively. The LiNbO_3_ powder CAS 12031-63-9 and CO_2_ from Sigma-Aldrich were used as the photocatalyst in this study. 

### 3.2. Sample Proportions

The concrete samples were fabricated in steel moulds with internal dimensions of 20 × 10 × 5 cm^3^. Cement, sand, and stone aggregate are mixed thoroughly with a mix ratio of 1 : 2 : 4 and water cement ratio of 0.5 and poured into the steel mould. Then the compaction was done by putting the steel moulds on a mechanical vibrating table. The surface of concrete was made smoothening with a glass plate. The concrete samples were removed from the moulds after 24 hours and putted into water for 28 days for proper curing. After curing, samples were allowed to dry in the air for a few hours. Dried sample was then coated with 0.75 mm LiNbO_3_ paste.

### 3.3. Equipment and Experimental Procedure

**Figure 3:**
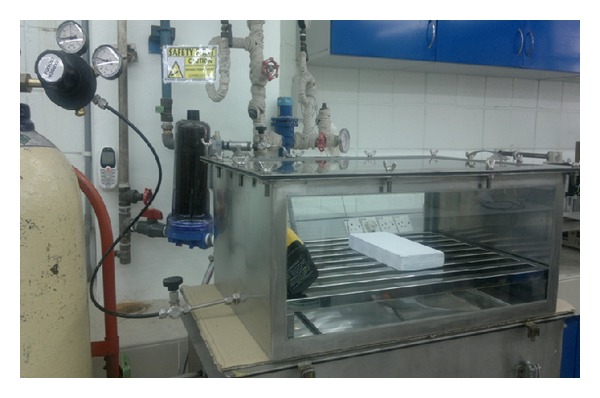
LiNbO_3_ coated concrete slab in reaction chamber.

The central part of the experimental setup used is a reaction chamber allowing a sample of size 20 × 10 × 5 cm^3^ to be fixed. All structural parts inside the box are to allow laminar flow of the gas along the sample surface and to make sure of the uniform distribution of CO_2_ gas. The reaction chamber was tested in experiments under reaction conditions before use and found to be air tight and nonreactive under prolonged UV irradiation. A fluorescent lamp of wavelengths 366 nm was used to supply photoirradiation to activate the photocatalyst. Two types of sensors were used to control the temperature and humidity.  Figure 3 shows the illustration of the reaction chamber and the test setup for artificial photosynthesis. LiNbO_3_ coated concrete block is placed in the reaction chamber. A reservoir of 30 mL of distilled water was then added in a tray before filling it with CO_2_ gas to create a 30 : 70 mix with air. An irradiation was carried out using a UV lamp or by natural sunlight; after irradiation, the vessel was allowed to cool and samples were collected from the water reservoir for analysis. 

## 4. Results and Discussion

### 4.1. FT-IR Analyses

FT-IR analyses were carried out with Bio-Rad FTS-185 (Digilab) equipment using Attenuated Total Reflexion (ATR) unit. The spectra were usually recorded in the range of 4000–400 cm^−1^ with 2 cm^−1^ resolution, and 32 scans were collected each time. 

**Figure 4:**
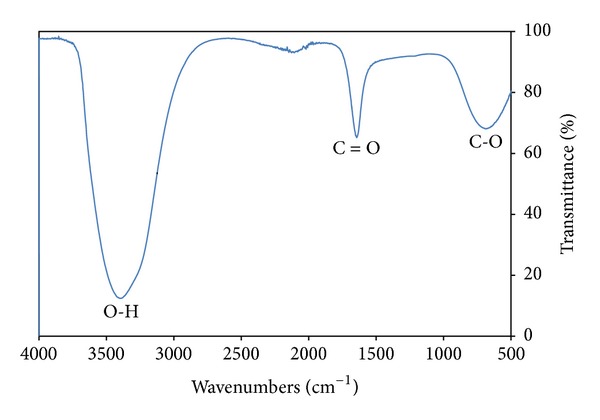
FT-IR spectra of water sample taken from reaction chamber after one day.

 Band assignments report available in literature are deducted from spectra performed in diffuse reflectance. In the present work, spectra acquisition is performed in ATR. A shift of about 3 wave number units is noted in spectra (Figure 4).

Nevertheless the band assignments are in close agreement with those previously reported [28–30]. The FT-IR data for band assignments are presented in Table 2. The broad bands at 3401.2 cm^−1^, 3402.3 cm^−1^, and 3401.7 cm^−1^ are due to the stretching O–H bond from HCOOH. The asymmetric stretching bands at 1644.3 cm^−1^, 1645.2 cm^−1^, and 1644.8 cm^−1^ are due to C=O bond in HCOOH. The wide bands at 687.1 cm^−1^, 685.3 cm^−1^, and 680.7 cm^−1^ are due to C–O bond in HCOOH.

### 4.2. HPLC Analyses

The HPLC of sample was performed on ZORBAX ECLIPS C18, 150 mm × 4.6 mm, 5 *μ* with a flow rate 0.6 mL/min keeping column temperature at 40°C. 

**Figure 5:**
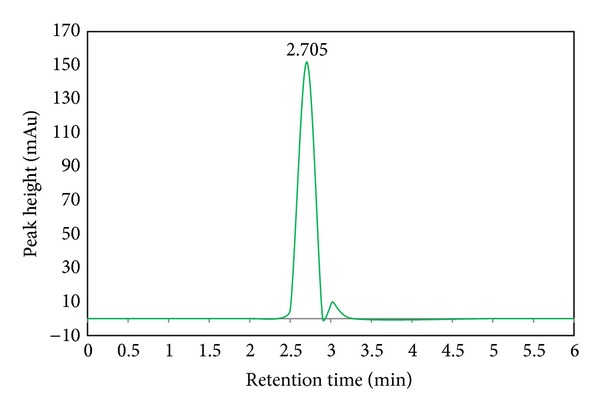
HPLC of sample taken from reaction chamber.

The mobile phase consisted of 20 mM H_2_SO_4_ in acetonitrile and detection of formic acid was performed by injecting 20 *μ*L of sample solution into a UV detector of wavelength 220 nm. The retention time (*R*_*t*_) of main peak was 2.6 min under these conditions [31]. The main peak of standard formic acid solution appears at the retention time (*R*_*t*_) 2.69 min. For comparison with the standard solution, spectrum of sample solution was taken at the same conditions. Each spectrum (Figure 5) gives the peak at the same retention time that indicates the presence of HCOOH. 

### 4.3. SEM Observations

**Figure 6:**
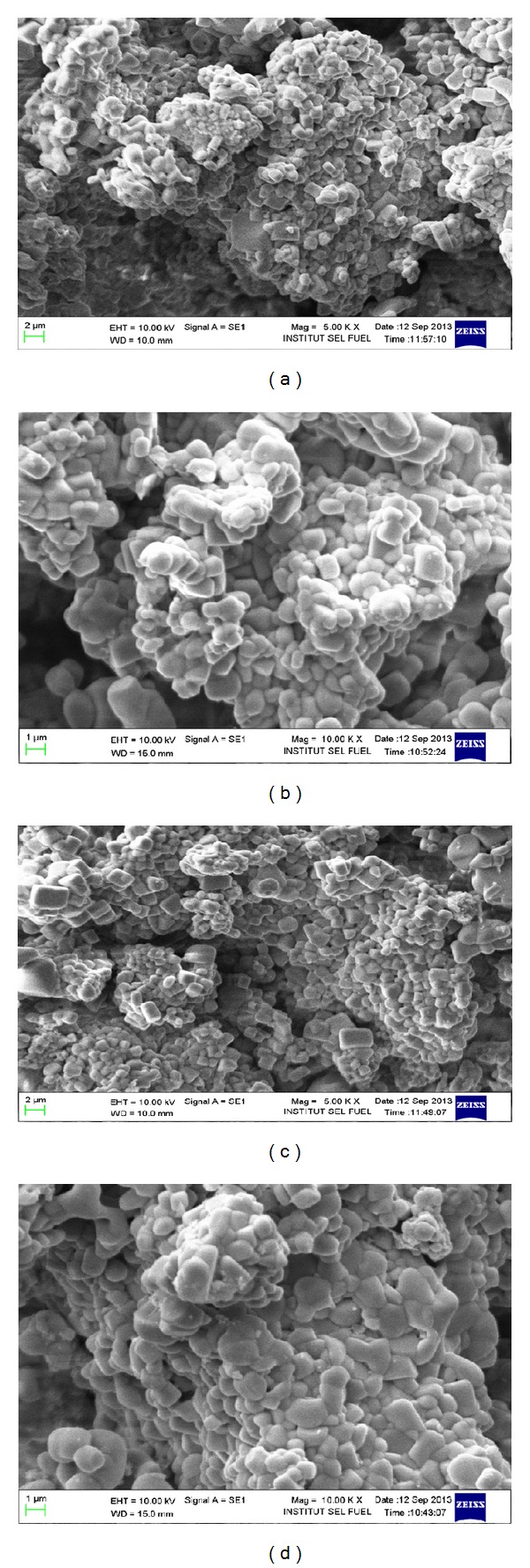
(a) SEM photomicrograph of the LiNbO_3_ before photocatalytic reaction on the specimen's surface. (b) SEM photomicrograph of the LiNbO_3_ before photocatalytic reaction on the specimen's surface. (c) SEM photomicrograph of the LiNbO_3_ after photocatalytic reaction on the specimen's surface. (d) SEM photomicrograph of the LiNbO_3_ after photocatalytic reaction on the specimen's surface.

Figures 6(a) and 6(b) illustrated the scanning electron microscope (SEM) photomicrograph of the LiNbO_3_ before photocatalytic reaction on the specimen's surface. The morphology of the particles is round and systematic structure particles. This is due to the carbonation of concrete that enhances the possibility of artificial photosynthesis. On the other hand microstructure of LiNbO_3_ particles is changed after the photocatalytic reaction. The SEM photomicrograph of the LiNbO_3_ after photocatalytic reaction on the specimen's surface is shown in Figures 6(c) and 6(d). Larger and darker agglomerates were appeared the microstructure of LiNbO_3_. A net decrease in porosity is also observed: pores are progressively filled due to the carbonation of concrete material that increases the contact of CO_2_ with photocatalyst.

### 4.4. EDX Analyses

**Figure 7:**
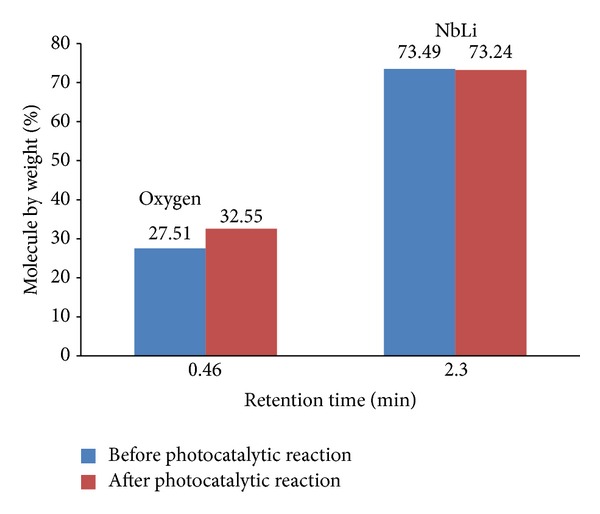
EDX analysis performed before and after photocatalytic reaction on specimens surface.

Chemical composition change was expected from energy-dispersive X-ray (EDX) analysis.  Figure 7 represents EDX analysis performed before and after photocatalytic reaction on specimens surface. Twenty punctual analyses are performed for each area. Expected change of chemical composition is observed. Figure shows that there is an increase in the amount of oxygen after photocatalytic reaction. 

The amounts of O and NbL are 27.51 and 73.49% by weight before photocatalytic reaction and the amounts of O and NbL, are 32.55 and 73.24% by weight after photocatalytic reaction which indicates the occurrence of artificial photosynthesis on specimens' surface.

## 5. Conclusions

This paper focuses on the application of LiNbO_3_ as photocatalytic material in concrete blocks. The addition of LiNbO_3_ in building materials adds an additional property to the concrete by creating artificial photosynthesis, which is the best way to convert CO_2_ into O_2_ and HCOOH and simultaneously reduce global CO_2_ level. This work demonstrates the impact of the photocatalytic reactions and shows LiNbO_3_ to be an exciting new material for use as a photocatalyst in construction. 

## Figures and Tables

**Table 1:** Key reaction of artificial photosynthesis with redox potential [10].

Reactions	*E*°, *V* versus SHE
CO_2_ + e^−^ → CO_2_^−^	−1.90
CO_2_ + 2e^−^ → CO_2_^2−^	−1.45
CO_2_ + 2e^−^ + 2H^+^ → HCOOH	−0.22
2H_2_O → O_2_ + 4H^+^ + 4e^−^	1.23
H_2_O → •OH + H^+^ + e^−^	2.10

**Table 2:** FT-IR characterization of sample.

Band assignments	Sample after one day (cm^−1^)	Sample after two days (cm^−1^)	Sample after three days (cm^−1^)
O–H	3401.2 s, b	3402.3 s, b	3401.7 s, b
C=O	1644.3 s	1645.2 s	1644.8 s
C–O	687.1 w	685.3 w	680.7 w
